# Factors associated with early initiation of breastfeeding among Nepalese mothers: further analysis of Nepal Demographic and Health Survey, 2011

**DOI:** 10.1186/s13006-014-0021-6

**Published:** 2014-12-05

**Authors:** Mandira Adhikari, Vishnu Khanal, Rajendra Karkee, Tania Gavidia

**Affiliations:** Women’s Health Project, Population Services International, Nawalparasi, Nepal; Independent Consultant, Kathmandu, Nepal; School of Public Health and Community Medicine, BP Koirala Institute of Health Sciences, Dharan, Nepal; Centre for International Health, Curtin University, Perth, Australia; School of Public Health, Curtin University, Perth, Australia

**Keywords:** Breastfeeding, Breastfeeding initiation, Determinants, Nepal

## Abstract

**Background:**

Timely initiation of breastfeeding has been reported to reduce neonatal mortality by 19.1%. The World Health Organisation recommends early initiation of breastfeeding i.e. breastfeeding a newborn within the first hour of life. Knowledge on the rate and the determinants of early initiation of breastfeeding may help health program managers to design and implement effective breastfeeding promotion programs. The aim of this study was to determine the rate and the determinants of early initiation of breastfeeding in Nepal.

**Methods:**

This study used the data from Nepal Demographic and Health Survey (NDHS) 2011 which is a nationally representative sample study. Chi square test and multiple logistic regression analysis were used to examine the factors associated with early initiation of breastfeeding (within one hour of birth).

**Results:**

Of 4079 mothers, 66.4% initiated breastfeeding within one hour of delivery. Mothers with higher education (Odds Ratio (OR) 2.56; 95% CI : 1.26, 5.21), mothers of disadvantaged Janjati ethnicity (OR 1.43; 95% CI : 1.04, 1.94), mothers who were involved in agriculture occupation (OR 1.51; 95% CI : 1.16, 1.97), mothers who delivered in a health facility (OR 1.67; 95% CI : 1.25, 2.23), whose children were large at birth (OR 1.46; 95% CI : 1.07, 1.99) were more likely to initiate breastfeeding within the first hour of child birth.

**Conclusions:**

Results suggest that two thirds of children in Nepal were breastfed within the first hour after birth. Although there was a higher prevalence of early initiation of breastfeeding among mothers who delivered in health facilities compared to mothers who delivered at home, universal practice of early initiation of breastfeeding should be a routine practice. The findings suggest the need of breastfeeding promotion programs among the mothers who are less educated, and not working. Such breastfeeding promotion programmes could be implemented via Nepal’s extensive network of community-based workers.

## Background

There are several proven benefits of early and exclusive breastfeeding to mother and infants. Colostrum contains a number of immunoglobulin and nutritious factors which protect the newborn from short and long term illnesses [1]. The World Health Organization (WHO) recommends that all neonates be breastfed within one hour of birth [2].

Around the world, early initiation of breastfeeding, breastfeeding within one hour of birth, has been reported to reduce neonatal mortality by 19.1-22% [1,3,4]. This is particularly important in resource-poor countries and those with high prevalence of communicable disease, as early initiation of breastfeeding is associated with protection against diarrhea, and also increases the likelihood of longer duration of exclusive breastfeeding, which helps with pregnancy-spacing, and saves the mothers resources in terms of money spent on formula and the long terms health effects of breastfeeding such as cognitive advantage [5]. Despite the strong evidence supporting immediate and long term health benefits of early initiation of breastfeeding and the efforts from the national governments and international agencies, early initiation of breastfeeding in South Asia remains low with the varying rate between the countries: 36.4% in India [6], 24% in Bangladesh [7] and 8.5% in Pakistan [8]. In 1991, the Baby Friendly Hospital Initiative (BFHI) was implemented by the WHO and United Nations Children’s Fund (UNICEF) [9]. The initiative aims to create a breastfeeding supportive environment in order to ensure newborns get the best start to life. The global initiative recommends 10 steps of successful breastfeeding; of which the first four steps are directly linked to early initiation [10].

In Nepal, the BFHI was first implemented in 1994, in 22 hospitals across the country, and while its effectiveness has not been evaluated nationally, internationally, the initiative has been shown to have a positive effect on breastfeeding rates [11]. At the community level, there are a number of health programs which have promoted the timely initiation and exclusive breastfeeding based on the National Nutrition Policy 2004 [12]. Notably, the Female Community Health Volunteers (FCHVs), a network of women who are trained to assist and counsel community members, particularly mothers, on pregnancy, perinatal, and newborn health practices, play an essential role in the delivery strategy of infant and young feeding interventions in Nepal [13]. More recently, FCHVs have been engaged in Community Based Newborn Care Package (CB-NCP), which aims to promote essential and immediate care to newborns and life saving care to those with special needs (low birth weight, birth asphyxia, hypothermia, infection) [14]. Given that nationally, 65% of deliveries occur at home, without the assistance from a trained provider, community-based initiatives and health workers play an important role in shaping breastfeeding behaviours. In Nepal, these networks of community health workers and volunteers are said to effectively reach mothers, including those who give birth at home, and in the hardest to reach communities.

Globally, various studies have shown that factors specific to the mother are associated with timely initiation of breastfeeding such as maternal education [6,15-18], and occupation [6,15,16]; the economic status of their household [16,19], and place of residence (rural vs. urban) [20,21] as being important factors in early initiation of breastfeeding. In addition, the use of antenatal care (ANC) has also been found to be positively associated with timely initiation of breastfeeding [6,16,22].

The 2006 Nepal Demographic and Health Survey (NDHS) reported that nationally, 35.4% of mothers initiated breastfeeding within one hour of childbirth [23]. However, at the district level, this figure varies significantly. A study from Kaski district of Western Nepal [24] reported a higher rate (57.9%) of early initiation, while a study from the Sarlahi district of Southern Nepal [3] reported a much lower rate of 3.4%. Factors associated with early initiation of breastfeeding have not been previously reported based on large nationally representative studies. The objective of this study was to determine the rate and the determinants of early initiation of breastfeeding in Nepal using the 2011 NDHS dataset. Results from this study could help to design and implement breastfeeding promotion programs to improve nutrition and survival of children in Nepal.

## Methods

This study used the data from the Nepal Demographic and Health Survey 2011, which is a nationally representative study [25]. This survey used two stage cluster sampling based on the enumeration areas and households samples. In the first stage, enumeration areas were selected. In the second stage, households were selected from each enumeration areas. A total of 289 enumeration areas (Rural-194; Urban-95) were selected. Details of sampling design, sampling frame, list of questionnaire are reported in publicly available report of NDHS 2011 [25]. The information of the mother-child pair of last born child within three years prior to the survey was included in the study.

### Definition of variables

#### Outcome variable

The outcome variable for this study was early initiation of breastfeeding defined as breastfeeding within the first hour of birth [2]. In the 2011 NDHS, mothers were asked; *when did you start breastfeeding to (Child’s NAME) after birth?* [25]. Responses were recorded in number of hours or days. The available responses of the question were recoded into: early initiation (within one hour), and late initiation (after one hour). We used the term early initiation to indicate timely initiation according to the WHO recommendation [2].

#### Independent variables

The independent variables used in this study were selected based on the previous studies in other countries [20] and available data on the NDHS 2011 [25,26]. Mother’s education recorded as no education, primary, secondary (grade 10) and higher secondary (grade 11 and above). Maternal reported occupation was re-categorised into not working (not working and no specific occupation), agriculture (paid and unpaid agricultural work) and paid work (office, business, clerical, manual) based on previously published literature [27]. Number of antenatal care (ANC) visits was recorded as a continuous variable and recoded into no ANC visit, 1–3 ANC visits, 4 or more ANC visits, with 4 or more ANC visits being the recommended number of visits by Ministry of Health and Population, Nepal [13]. Place of delivery was categorized as Health facility (Sub-Health post, Health post, Primary health care centre, and private health facilities); and home. Ethnicity was recorded as advantaged (Brahmins, Chhetris, Thakuris and Sanyashi, Gurung, Newar), disadvantaged Janjatis (indigenous castes) and disadvantaged Dalits. Advantaged groups occupy the highest rank in Nepal’s social hierarchy, while the Dalit ethnicity occupies the lowest, with Janjati placed in the middle [26]. The wealth quintile variable, calculated by principal component analysis of available assets during survey, was recoded into the lowest 40% as poor; middle 40% as average; and the upper 20% as rich. Re-categorisation was based on the similarities among the quintiles and based on previously published studies [26,28]. Birth order was recoded into first; second or third; and fourth or more. Mother’s perceived size of baby was recorded in the NDHS 2011 as very small; smaller than average; average; larger than average and very large which was further recoded into small (very small and smaller than average); average and large (larger than average and very large). For administrative purposes, Nepal is divided into five regions, namely: Eastern, Central, Western, Mid-western and Far-western region. The Far and Mid -western regions of Nepal are generally considered remote, difficult to reach and the most underdeveloped of all the regions, primarily due to their distance from the most modern and urbanised Central region and their difficult terrain, which is consequently credited for their limited road networks, poorly maintained roads and weak public transport system [29,30]. The result is that the residents of the Far and Mid -western regions are highly marginalised from the reach of social services, such as water and sanitation infrastructure, with the vast majority found to live in poverty, and experiencing some form of food insecurity [25,29]. A further division of country is three ecological regions based on altitude, namely: mountains (the northern Himalayan rage, mostly covered with snow; more than 3000–8850 meters), hills (middle section of hills; 600–3,000 meters altitude), and terai (the southern plain area; below 600 meters) [31].

### Statistical analysis

The rate of early initiation of breastfeeding and distribution by different independent variables was reported as percentage. Chi square tests (χ2) were performed to evaluate the association of the independent variables with the early initiation of breastfeeding. Statistically significant variables in Chi square test were further examined using multiple logistic regression analysis. Unadjusted and adjusted odds ratios with their 95% confidence interval (CIs) were reported. A p-value <0.05 was considered statistically significant. Statistical analysis was performed by using Statistical Package for Social Sciences (SPSS) version 20. The analysis accounted for the sample design and sample weight using Complex Sample Analysis method [32].

### Ethics statement

The NDHS 2011 was approved by ethics committee in the Nepal Health Research Council, Nepal, and the human research ethics committee in ICF Macro International [25]. Participants provided written consent (or thumb print) for the study. Mothers or care takers provided consent for children. The data was made publicly available after removing personal identifiers.

## Results

Table 1 presents the socio-demographic characteristics of the mothers. A total of 4079 mothers with last born children within three year preceding the survey were included in the study. The majority (71.1%) of the women were in the 20–29 years age group. Four in every ten mothers were illiterate. The majority (61.3%) were involved in the agriculture sector, either as paid or unpaid (subsistence) farmers. More than half (52.7%) reported attending the recommended four or more ANC visits, with approximately one third (32.3%) attending 1–3 ANC visits. Six in every ten (60.4%) mothers reported a home delivery for their most recent birth, and almost half of the study population (48.8%) was classified as poor.

**Table 1:** **Rate (%) of initiation of breastfeeding within one hour of childbirth by demographic and socioeconomic characteristics, Nepal 2011 (N = 4079)**

**Factor**	**# Total N [%]**	**Early initiation € n (%)**	**P-value**
**Maternal factors**			
**Mother’s age at pregnancy (n = 2518)**			0.006
15-19	245 (9.7)	211 (60.8)	
20-29	1791 (71.1)	1888 (68.5)	
30-34	282 (11.2)	454 (66.8)	
> = 35	200 (7.9)	308 (58.7)	
**Maternal education**			0.001
No education	1765 (43.3)	1130 (57.9)	
Primary	817 (20.0)	600 (70.0)	
Secondary	1225 (30.0)	927 (74.6)	
Higher	272 (6.7)	204 (73.6)	
**Mother’s occupation**			0.001
Not working	965 (23.7)	618 (59.1)	
Agriculture	2502 (61.3)	1775 (68.0)	
Working (paid)	612 (15.0)	468 (74.2)	
**ANC visit (Times)**			0.001
No ANC visit	611 (15.0)	382 (59.4)	
1-3	1317 (32.3)	885 (61.8)	
4 or more	2151 (52.7)	1594 (71.7)	
**Place of delivery**			0.002
Home	2464 (60.4)	1670 (63.9)	
Health facility	1615 (39.6)	1191 (70.5)	
**Sociodemographic factors**			
**Ethnicity**			0.048
Relatively advantaged	1955 (47.9)	1359 (66.3)	
Relatively disadvantaged (Janjati)	1410 (34.6)	1022 (69.8)	
Relatively disadvantaged (Dalit)	714 (17.5)	480 (58.8)	
**Religion**			0.188
Hindu	3480 (85.3)	2464 (67.3)	
Others	599 (14.7)	397 (61.9)	
**Wealth quintile**			0.073
Poor (Lower 40%)	1992 (48.8)	1375 (65.3)	
Middle (Middle 40%)	1416 (34.7)	513 (62.3)	
Rich (Upper 20%)	671 (16.5)	973 (70.4)	
**Child related factors**			
**Sex of child**			0.098
Male	2192 (53.7)	1563 (68.0)	
Female	1887 (46.3)	1298 (64.9)	
**Birth order**			<0.001
First	1248 (30.6)	854 (64.0)	
Second or third	1847 (45.3)	1367 (70.5)	
Fourth or more	984 (4.1)	640 (61.5)	
**Size of baby (n = 3945)**			0.035
Average	784 (19.2)	1808 (65.4)	
Small	2576 (63.2)	477 (64.2)	
Large	715 (17.5)	574 (71.7)	
**Contextual factors**			
**Place of residence**			0.816
Urban	897 (22.0)	638 (67.0)	
Rural	3182 (78.0)	2223 (66.3)	
**Development region**			<0.001
Eastern	958 (23.5)	679 (69.7)	
Central	854 (20.9)	504 (54.4)	
Western	643 (15.8)	456 (70.5)	
Mid -Western	896 (22.0)	642 (71.4)	
Far-Western	728 (17.8)	580 (79.9)	
**Ecological region**			0.017
Mountain	742 (18.2)	529 (70.5)	
Hill	1656 (40.6)	1185 (70.4)	
Terai/Plain	1681 (41.2)	1147 ( 62.7)	

€ The percentages presented for the early initiation of breastfeeding are the weighted and cluster sampling adjusted percentage which differs from the crude percentage. The number of missing values may vary for each variable. # the number and percent reported are unweighted for the independent variables and their sub class to facilitate the reading as weighted count (frequency) will be in decimal points generated by the software.

A total of 2861 women (weighted proportion 66.4%; 95% CI: 62.9%- 69.7%; design effect 5.5) had initiated breastfeeding within one hour of delivery. Mother’s age at pregnancy, maternal education, maternal occupation, number of ANC visits, place of delivery, ethnicity, birth order, baby size, development region and ecological region were statistically significant in univariate analysis (Table 1). The proportion of babies receiving early initiation of breastfeeding increased with increasing maternal education. About 70.5% of mothers who delivered in health facilities initiated breastfeeding within the first hour after delivery, compared to 64% of mothers who delivered at home. A higher proportion of infants of Janjati ethnicity were breastfed within one hour.

Table 2 presents the result of multiple logistic regression analysis of the significant variables in the univariate analysis. Maternal education, ethnicity, mother’s occupation, place of delivery, size of baby, and ecological region were significantly associated with early initiation of breastfeeding. Education had a positive effect on early initiation of breastfeeding showing that mothers having higher education (OR 2.56; 95% CI: 1.26, 5.21), secondary education (OR 1.91; 95% CI: 1.34, 2.73) and primary education (OR 1.69; 95% CI: 1.25, 2.28) were more likely to initiate early breastfeeding than women with no education. Mothers belonging to relatively disadvantaged Janjatis (OR 1.43; 95% CI: 1.04, 1.94) were more likely to initiate breastfeeding than relatively advantaged ethnic groups. Mothers who were involved in agriculture (OR 1.51; 95% CI: 1.16, 1.97) were more likely to initiate breastfeeding during the first hour of childbirth. Health facility delivery had a positive influence on early initiation of breastfeeding, with the infants who were delivered in health facilities were more likely (OR 1.67; 95% CI: 1.25, 2.23) to be breastfed within one hour than their counterparts born at home. The mothers who reported large sized babies at the time of birth (OR 1.46; 95% CI: 1.07, 1.99) were more likely to initiate breastfeeding earlier than those mother who reported average sized babies. Mothers from Far-western region (OR 3.29; 95% CI: 2.12, 5.09), Mid-western region (OR 2.06; 955 CI: 1.44, 2.95), Eastern region (OR 1.63; 95%:1.11, 2.41) and Western region (OR 1.53; 95% CI: 1.03, 2.29) were more likely to initiate breastfeeding within first hour compared to those from the Central region.

**Table 2:** **Factor associated with early initiation of breastfeeding in Nepal 2011**

**Factor**	**Crude OR (95% CI)**	**P-value**	**Adjusted OR (95% CI)**	**P-value**
**Mother’s age at pregnancy**		0.010		0.105
15-19	1.00		1.00	
20-29	1.06 (0.72, 1.57)		1.00 (0.69, 1.47)	
30-34	0.90 (0.56, 1.44)		0.94 (0.54, 1.61)	
> = 35	0.58 (0.37, 0.86)		0.68 (0.43, 1.08)	
**Maternal education**		0.001		0.009
No education	1.00		1.00	
Primary	1.74 (1.33, 2.27)		1.69 (1.25, 2.28)	
Secondary	2.13 (1.67, 2.71)		1.91 (1.34, 2.73)	
Higher	2.02 (1.44, 2.83)		2.56 (1.26, 5.21)	
**Ethnicity**		0.0 5		0.015
Relatively advantaged	1.00		1.00	
Relatively disadvantaged (Janjati)	1.18 (0.86, 1.61)		1.43 (1.04, 1.94)	
Relatively disadvantaged (Dalit)	0.73 (0.50, 1.05)		0.85 (0.58, 1.23)	
**Mother’s occupation**		<0.001		0.007
Not working	1.00		1.00	
Agriculture	1.17 (1.15, 1.90)		1.51 (1.16, 1.97)	
Working (paid)	1.99 (1.46, 2.72)		1.45 (0.99, 2.10)	
**ANC visit (number)**		<0.001		0.212
No ANC visit	1.00		1.00	
1-3	1.11 (0.87, 1.43)		1.22 (0.90, 1.64)	
4 or more	1.73 (1.30, 2.32)		1.25 (0.88, 1.78)	
**Place of delivery**		0.002		0.001
Home	1.00		1.00	
Health facility	1.35 (1.12, 1.63)		1.67 (1.25, 2.23)	
**Birth order**		0.05		0.965
Fourth or more	1.00		1.00	
Second or third	1.50 (1.24, 1.80)		0.99 ( 0.80, 1.42)	
First	1.11 ( 0.89, 1.42)		0.99 (0.756, 1.31)	
**Size of baby**		0.032		0.002
Average	1.00		1.00	
Small	0.95 (0.74, 1.21)		0.76 (0.54, 1.06)	
Large	1.34 (1.06, 1.68)		1.46 (1.07, 1.99)	
**Development region**		0.002		0.001
Central	1.00		1.00	
Eastern	1.93 (1.29, 2.88)		1.63 (1.11, 2.41)	
Western	2.01 (1.33, 3.03)		1.53 (1.03, 2.29)	
Mid -western	2.09 (1.38, 3.17)		2.06 (1.44, 2.95)	
Far-western	3.32 (2.19, 5.05)		3.29 (2.12, 5.09)	
**Ecological region**		0.038		0.217
Terai/plain	1.00		1.00	
Mountain	1.42 (1.02, 1.97)		1.19 (0.84, 1.70)	
Hill	1.42 (1.06, 1.87)		1.19 (0.90, 1.56)	

Independent variables entered in the initial model: All variables included in the model are shown in the table.

## Discussion

This study found a higher rate (66.4%) of early initiation of breastfeeding compared to previous studies done in the Kaski district, Western Nepal (57.9%) [24], NDHS 2006 (35.4%) [23], Central Nepal (63.0%) [33] and Southern Nepal (3.4%) [3]. Similarly, the rate is higher when compared with previous studies from India (36.4%) [6]; Bangladesh (24%) [7]; and Pakistan (8.5%) [8] which have comparable socioeconomic condition and cultural practices. The higher rate of early initiation of breastfeeding reported in this study can be attributed to increased access to skilled birth attendants, effective interventions for child and neonatal survival strategies and Nepal’s continued commitment to promote breastfeeding through a number of newborn and child health programs. Nepal’s multilevel approach to addressing early initiation of breastfeeding should perhaps be largely credited for the high rate of early initiation of breastfeeding. At the hospital level, the BFHI promotes early breastfeeding initiation, exclusive breastfeeding, and breastfeeding duration, while beyond the hospital setting, the involvement of FCHVs in the delivery of CB-NCP at the community level, provides guidance and support for mothers, especially underserved mothers, to adopt or improve breastfeeding practices [34]. For instance, a recent evaluation of CB-NCP also documented that this community-based program was successful in increasing the rate of early initiation of breastfeeding (from 44% to 75%) [14].

Maternal education, ethnicity, mother’s occupation, place of delivery, size of baby and development region were all found to be significantly associated with early initiation of breastfeeding. In this study, health facility delivery was positively associated with early initiation. This is of no surprise given that health professionals are immediately present to advice and inform the mother on appropriate feeding practices [35,36]. However, 65% of births in Nepal occur outside of health facilities, and are mostly attended by senior women and relatives; with the presence of skilled birth attendant during home deliveries are reported to be very low at only 2% [37]. In such situation, newborns may be put to breastfeeding only after bathing, a common practice in Nepal [33]. Similar to our finding, studies from Sri Lanka, Vietnam and China [16,36,38] have also reported that skilled birth attendants were likely to follow the national guidelines on breastfeeding and hence support postpartum mothers for timely initiation of breastfeeding [35,36]. While the current rate of early initiation both in health facility (70.5%) and home (63.9%) were significantly higher than those reported during the 2006 NDHS (health facility: 39.9%; home: 34.3%) [23], a significant proportion (30%) of children born in health facilities were not breastfed within the first hour after birth. Admission into neonatal intensive care unit or conditions of the mother such as severe bleeding during delivery, or caesarean section have been previously found as barriers to early breastfeeding [39]. Such maternal and newborn conditions may also account for some of the observed delay in early initiation of breastfeeding in health facilities in Nepal. This would suggest that more efforts are needed on adapting the BFHI to especial neonatal and maternal emergency conditions. More recently, overcrowding in public hospitals has been suggested as a plausible driver for the decline in the quality of services and reduced time for health provider-client interactions [13]. This may also provide an explanation for delays in the initiation of breastfeeding.

Similar to previous findings from India [6] and England [15], this study showed a positive association of maternal education and early initiation of breastfeeding. The effect of education may be explained by the likelihood that educated mothers are more able to receive and understand health promotion messages. They are also more likely to deliver in health facilities or to obtain the skilled assistance during delivery [40].

Maternal occupation was also found to have a significant association with early initiation of breastfeeding, with mothers who were not working less likely to initiate early breastfeeding than the mothers working in agriculture. Women engaged in paid work may have more access to financial resources, and due to attitudes related to modernity and urbanisation, may be more accepting of infant formula. Equally, working women may also be able to afford cesarean deliveries, a factor previously associated with delays in early initiation of breastfeeding. Results from the 2011 NDHS indicated that a higher proportion of women from richest wealth group (14.1%) have cesarean deliveries than the national average (4.6%) [25]. An alternative explanation for this effect of occupation on early initiation of breastfeeding may be that mothers who reported themselves as ‘not working’ may be less educated, deliver in home, and living in socially disadvantaged areas, all of which are the proven risk factors for delaying timely initiation of breastfeeding [21]. The association on maternal employment and initiation of breastfeeding has not been uniform. For instance, in a Chinese study, Wang et al. [19] reported that part-time working mothers were more likely to start early breastfeeding, while in Bangladesh, a study showed that the employment status of the mother was not associated with the early initiation of breastfeeding [7]. Future studies could explore the effect of employment on timely initiation of breastfeeding in Nepal.

Ethnicity is a strong cultural identity which define how and what mothers do in terms of their health related behaviors including child feeding practices [41]. In our study, the mothers from disadvantaged Janjati groups were more likely to initiate breastfeeding in the first hour of childbirth. Disadvantaged Janjati groups are more likely to be from the households with lower wealth status [33]. Such poor status may be a factor to initiate breastfeeding earlier as that remains the only option for infant feeding. Further qualitative studies could also be carried out to explore the reason for higher early initiation of breastfeeding by Janjati mothers.

Mothers who reported the size of their baby as ‘large’ at the time of birth, were more likely to initiate breastfeeding early, a finding similar to those from India and Brazil [6,22]. Mothers and health workers may perceive that the large sized babies are fully mature and possess good functioning of suction and deglutition. They may also perceive that the child is healthy; therefore, may lead to initiating early breastfeeding [22]. However, it is small sized infants who need more care and breastfeeding immediately after birth to support and maintain their body temperature, and facilitate proper weight gain after birth. This finding suggests that mothers with small sized infants need more attention and support for initiating breastfeeding.

Development region was found to be a predictor of early initiation of breastfeeding. Babies from Far-western region, Mid-western, Eastern and Western were more likely to receive breastfeeding early than their Central region counterparts. Similar to our findings, a previous study from Southern part of Central region also reported a very low rate (3.4%) of early initiation [3]. A number of previous studies have suggested a regional difference in child feeding practices in Nepal [20,26,42]. Mid and Far-western Nepal is characterized by difficult terrain, higher poverty status, lower socioeconomic development indicators which may restrict the availability of infant formula and television advertisements to promote infant formulas. These factors may leave breastfeeding as the only option for mothers to feed infants [43]. This finding also confirms that the rate of early initiation varies significantly in the five regions of Nepal.

The findings of this study are of public health significance. This study showed that a significant proportion of mothers who deliver in health facilities did not initiate breastfeeding within the first hour; even though health facility delivery was associated with a higher rate of early initiation. This finding suggests that mothers need further support, and encouragement to initiate breastfeeding such as those recommended by the BFHI [44]. A further scaling up of the ten steps to successful breastfeeding to all hospitals of Nepal is likely to have a positive impact. Adoption of some of the recommendations of the BFHI have been found to be effective at lower level health facilities such as birthing centres [45], and there is further opportunity to scale up such recommendations in these settings in Nepal. This is important in Nepal as birthing centres are currently serving as important delivery places for the most vulnerable women. This study further highlights the need of breastfeeding promotion programs amongst mothers who are less educated, unemployed, those who give birth at home, and those from the Central regions of Nepal. Peer education has a significant impact on early initiation [46]. To promote early initiation in Nepal, women can be educated during pregnancy and postpartum period through existing Female Community health Volunteer (FCHV) network [13].

This study is based on a nationally representative study and large sample size. The questionnaire and the study methodology are validated. The analysis accounted for the study design and sample weight [25]. A major limitation of this study is cross sectional design with possible retrospective recall bias.

## Conclusion

Two in every three mothers had initiated breastfeeding within one hour of childbirth. The factors associated with the early initiation of breastfeeding were the place of delivery, maternal education and occupation, baby’s size at birth and developing regions. Given that Nepal’s rate of neonatal mortality, stunting, and wasting have remained relatively unchanged over the last five years, the promotion of early initiation of breastfeeding is likely to have positive impact in neonatal and infant survival. Breastfeeding awareness campaigns or counseling through Nepal’s existing strong network of FCHVs, and health workers to pregnant women focusing on less educated, unemployed, and those from the central region may be helpful to improve early initiation of breastfeeding in Nepal.
